# Discordance between *in vitro* susceptibility and clinical response in early-onset neonatal sepsis caused by *Morganella morganii*: a case report in an extremely preterm infant

**DOI:** 10.3389/fped.2026.1859505

**Published:** 2026-07-30

**Authors:** Eman Akram Saleh Omari, Yousef Khaleel Albow, Bashaer Imad Iwaiwi, Motee Abuawwad

**Affiliations:** 1 Faculty of Medicine, Al-Quds University, Jerusalem, Palestine; 2 Department of Pediatrics, Al-Ahli Hospital, Hebron, Palestine

**Keywords:** antibiotic sensitivity testing, case report, early-onset neonatal sepsis (EONS), *Morganella morganii*, premature neonate

## Abstract

Early-onset neonatal sepsis (EONS) caused by *Morganella morganii* is rare, particularly in extremely preterm infants, and poses diagnostic and therapeutic challenges. We report a case of EONS in an extremely preterm neonate in whom blood cultures identified *Morganella morganii* with *in vitro* susceptibility to gentamicin, which was initiated as part of the empirical treatment. Despite documented antimicrobial sensitivity, the infant showed no significant clinical improvement. Consequently, antibiotic therapy was modified based on both susceptibility testing and the evolving clinical picture, resulting in marked clinical recovery. This case highlights that antimicrobial susceptibility results should be interpreted in conjunction with the patient's clinical response and that treatment decisions in neonatal sepsis should not rely solely on *in vitro* sensitivity patterns, particularly when dealing with uncommon pathogens.

## Introduction

EONS is a significant clinical condition predominantly affecting premature infants and represents a major cause of morbidity and mortality. It typically develops within 72 hours after birth due to infections acquired before or during delivery (1). The incidence of EONS varies across regions, influenced by healthcare systems and population characteristics, with studies reporting 31.3 cases per 1,000 live births (2, 3). The most common causative pathogenic organisms include Group B. Streptococcus and E. coli (4). *Morganella morganii* is a rare cause of neonatal sepsis (5). Its clinical significance lies in its intrinsic resistance to multiple antibiotics, including certain beta-lactams, which may complicate treatment and delay effective therapy (6). Here, we present a case of EONS in an extremely preterm infant caused by *Morganella morganii*, highlighting a rare pathogen of EONS and key challenges in diagnosis and selecting the appropriate antibiotics.

## Case presentation

A male neonate was delivered via normal vaginal delivery at 27 + 4 weeks of gestation to a 23-year-old primigravida who presented in spontaneous preterm labor. The antenatal course was notable for a treated urinary tract infection in the second trimester. The mother received treatment at another healthcare facility; however, detailed microbiological records, including urine culture results, antimicrobial susceptibility testing, and antibiotic therapy, were unavailable despite attempts to retrieve them. With otherwise normal ultrasounds. Birth weight was 1400 g. Apgar scores were 7 and 8 at 1 and 5 minutes, respectively. Due to prematurity and respiratory distress, the infant was admitted immediately to the neonatal intensive care unit (NICU).

**Figure 1:**
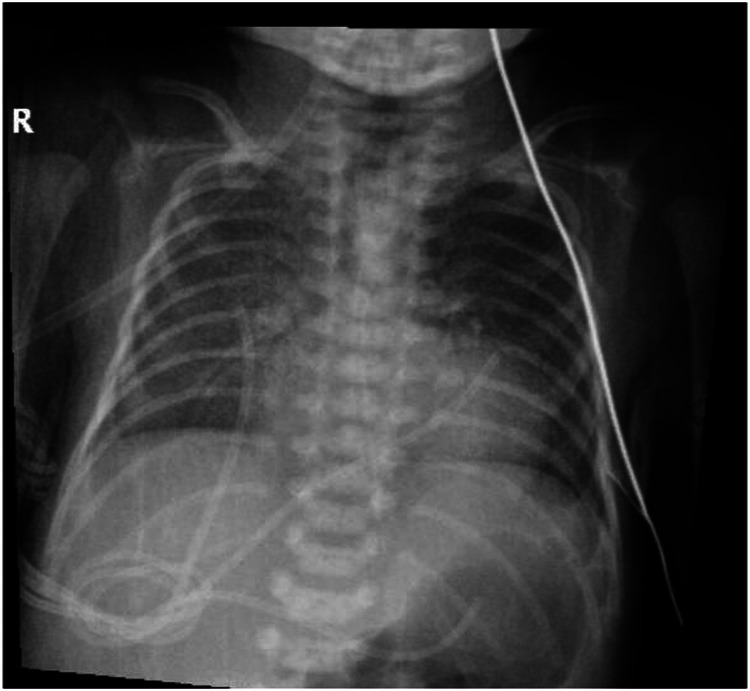
Representative de-identified chest radiographs demonstrating findings consistent with respiratory distress syndrome in an extremely preterm infant. The images provide clinical context for the respiratory course and illustrate the contribution of prematurity-related lung disease in addition to neonatal infection.

On admission (day 0 of life), the neonate exhibited signs of respiratory distress, including tachypnea, nasal flaring, grunting, and intercostal retractions. Chest radiography demonstrated findings consistent with respiratory distress syndrome (Figure 1). An umbilical venous catheter was inserted for vascular access. A lumbar puncture was attempted but was technically limited, resulting in an insufficient amount of cerebrospinal fluid for full analysis. Only a Cerebrospinal fluid (CSF) culture was performed, and it was negative.

Initial laboratory investigations revealed an elevated C-reactive protein (CRP) level of 27 mg/L. The white blood cell (WBC) count was 8.3 × 10³/µL with 67.7% neutrophils, and the platelet count was 183 × 10⁹/L, both within expected ranges for gestational age. Empirical intravenous antibiotic therapy with ampicillin and gentamicin was initiated. The gentamicin regimen was administered according to the local NICU protocol, which recommends extended-interval dosing for extremely preterm infants.

During the first days of admission, blood cultures grew *Morganella morganii*, which demonstrated *in vitro* susceptibility to gentamicin. The infant continued to experience respiratory difficulties and evolving laboratory abnormalities despite antimicrobial therapy. However, the respiratory course was likely multifactorial, reflecting both underlying respiratory distress syndrome associated with extreme prematurity and the systemic effects of neonatal infection. Therefore, persistent respiratory symptoms alone could not be attributed exclusively to uncontrolled infection.

Serial laboratory monitoring (Table 1) showed progressive leukocytosis, with WBC rising to 40 × 10³/µL accompanied by a left shift, along with a decline in platelet count to 135 × 10⁹/L, consistent with evolving sepsis.

**Table 1:** Timeline table of clinical course, investigations, and management.

Date/period	Clinical course	Work up/key results/management
Day 0	Date of birth	1. At Delivery: • Birth weight: 1400 g • GA: 27 + 4 weeks • Apgar score: 7/8 at 1 and 5 min, respectively. 2. Assessment: • Baby born vigorous, cried immediately, transferred immediately to NICU as a case of prematurity, RDS. 3. Plan: • R/O sepsis for further evaluation and management. • Baby intubated on NICU and given one dose of surfactant • CRP was 27. • Blood sample for culture was obtained. • Ampicillin- gentamicin was started • Caffeine was given
Day 1	Follow-up	1. Labs: • CRP 33, TSB 9, level5 of photo and level 8 of exchange , serum calcium 5.4, BUN 26, Cr 0.77. 2. Plan: • Baby extubated. • Start phototherapy • Make Ampicillin • Repeat TSB, CBC, Direct coombas test. • Follow-up BUN, Cr.
Day 2	Follow-up	1. Labs: • Ca: 6.7 • WBC: 40k 2. Plan: • Continue same Antibiotic
Day 3	Follow-up in the first hospital.	1. Blood culture result: • *Morganella morganii* susceptible to Gentamicin, Meropenem and Tazocin. 2. Labs: CRP: 10.7 WBC: 35.6k Hb: 12 Platelet count: 287k 3. Plan: • Continue same medication • Transfer to another facility for further evaluation
	Follow-up in the second hospital.	1. Initial evaluation: • CW 1200gm, CGA 28 weeks. • Admitted to the NICU • V/S: HR 143, RR 55, Temp 36.2, BP 70/49, SPO2 98% on 1L NC.
		• ABG: PH 7.29, PCO2 35.4, HCO3 17.2, BE −8. • Physical exam: grunting and in respiratory distress. open anterior frontanelle, • soft and lax abdomen, normal male genitalia, patent anus. • After stabilization, he weaned to room air and remained stable for more than 24 h. • TFU showed bilateral flaring. • Antibiotic switched to amikacin and tazocin for 4 days, then amikacin D/C and completed. • Tazocine-amikacin given for 14 days.
Day 11	Follow-up	1. Physical exam: • Developed repeated sudden brief desaturation and one apnea episode. 2. Plan: • connected to the nasal cannula.
Day 15	Follow-up	• LP was tried and CSF culture showed negative results.
Day 21	Follow-up	• Hepatitis B vaccine was given • Gauther test was done • Weaned to room air • developed apnea so continue on NC then remained stable on room air for 1 month
Day 34	Follow-up	• PRBCs was given due to anemia (Hb 8.8) • Control blood culture was done and show negative result
Day 52	Discharge	• Well and active • Discharge weight 2.25 kg • HC: 33 cm • Ht: 49 cm

GA, gestational age; NICU, Neonatal Intensive Care Unit; RDS, respiratory distress syndrome; CRP, C-reactive protein; HCO3-, bicarbonate; BE, base excess; LAC, lactate; BUN, blood urea nitrogen; Cr, creatinine; TSB, total serum bilirubin; WBC, white blood cells; Hb, hemoglobin; CW, current weight; CGA, corrected gestational age; HR, heart rate; BP, blood pressure; ABG, arterial blood gases; TFU, transfontanelle ultrasound; LP, lumbar puncture; CSF, cerebrospinal fluid; NC, nasal cannula; PRBCs, packed red blood cells; HC, head circumference; Ht, height.

Due to clinical deterioration and after consultation with the infectious disease team, antibiotic therapy was escalated to amikacin and piperacillin–tazobactam for 14 days, resulting in clinical improvement. A follow-up blood culture was obtained after initiation of amikacin and piperacillin–tazobactam therapy and demonstrated no bacterial growth, confirming microbiological clearance after treatment adjustment.

Due to respiratory distress shortly after birth, the infant required intubation and received one dose of surfactant for respiratory distress syndrome related to extreme prematurity. Following respiratory stabilization, the infant was extubated and transitioned to non-invasive ventilation with nasal continuous positive airway pressure (N-CPAP). The infant showed gradual respiratory improvement and was successfully weaned to room air by day 5 of life.

Additional management included phototherapy and intravenous immunoglobulin for hyperbilirubinemia. Enteral feeding was initiated and advanced according to NICU protocol. Caffeine therapy for apnea of prematurity was discontinued at a corrected gestational age of 34 weeks.

The infant demonstrated steady clinical improvement, achieving full oral feeding and appropriate weight gain. At discharge on day 52 of life, the neonate weighed 2250 g, with a head circumference of 33 cm. He was discharged on vitamin D and iron supplementation, with scheduled pediatric and developmental follow-up.

## Discussion

*Morganella morganii* is a rare cause of EONS, particularly in extremely preterm infants. While it is more commonly reported in adult nosocomial infections, in neonates, vertical transmission from maternal colonization appears to be the primary route of infection (7, 8). Maternal risk factors, such as urinary tract infections, chorioamnionitis, and prior antenatal antibiotic exposure, along with neonatal factors like prematurity, low birth weight, and male sex, increase susceptibility to EONS (1, 9).

A limitation of this case is the unavailability of maternal microbiological and antimicrobial treatment records, which limited further assessment of potential maternal risk factors.

### Diagnostic challenge

Neonatal sepsis diagnosis relies on a combination of several factors including: history, physical examination, laboratory investigations, and culture (10). In this case, limited lumbar puncture due to minimal handling was considered challenging, which restricted the ability to rule out other issues such as meningitis. Although meningitis was not observed in our case, four *Morganella morganii*-associated meningitis cases have been reported, emphasizing the importance of performing a lumbar puncture (11).

### Differential diagnosis

Clinical signs of M. morganii sepsis in neonates are nonspecific, resembling other bacterial infections such as meningitis or other neonatal respiratory conditions like Transient Tachypnea of the Newborn (TTN), and include fever, tachycardia, hypotension, and feeding difficulties (12, 20). The most common symptoms are respiratory distress, tachypnea, and perinatal depression (11). In our case, the newborn exhibited mild respiratory distress syndrome at birth and elevated CRP, which were likely indicative of sepsis. Physical examination alone cannot reliably differentiate between different types of serious neonatal infections, so a full evaluation is required. This includes blood, urine, and cerebrospinal fluid (CSF) cultures, along with a complete blood count and urinalysis (13).

### Therapeutic consideration

*Morganella morganii* possesses inducible chromosomal AmpC β-lactamases, conferring intrinsic resistance to aminopenicillins and early-generation cephalosporins. These resistance mechanisms may contribute to treatment failure and should be considered when selecting antimicrobial therapy (14).

Although *Morganella morganii* was sensitive to gentamicin (Tables 2, 3), the neonate showed no clinical improvement on empirical therapy: respiratory distress, recurrent use of mechanical ventilation, recurrent episodes of apnea, which may be explained by the organism's ability to develop resistance (6), illustrating that antimicrobial susceptibility alone does not reliably predict clinical response in high-risk neonates. Clinical deterioration despite appropriate empirical therapy necessitated adjustment of antibiotics to amikacin and piperacillin–tazobactam, after which the infant improved. Therapeutic drug monitoring for gentamicin and amikacin was not performed because serum antimicrobial concentration testing was not routinely available at our institution. Therefore, antibiotic exposure could not be assessed, which represents a limitation when interpreting the relationship between antimicrobial susceptibility results and clinical response. Gentamicin dosing recommendations for extremely preterm neonates vary among institutions. In our center, extended-interval dosing of approximately 4–5 mg/kg every 36–48 hours is routinely used.

**Table 2:** Serial laboratory findings and clinical course during hospitalization.

Day of life	CRP (mg/L)	WBC ( × 10³/µL)	Neutrophils (%)	Hemoglobin (g/dL)	Platelets ( × 10⁹/L)	Creatinine (mg/dL)	Clinical status
0 (Admission)	27	8.3	67.7	—	183	—	Respiratory distress; empirical ampicillin + gentamicin initiated
1	33	—	—	—	—	0.77	Persistent respiratory distress; phototherapy initiated
2	—	40.0	Left shift	—	135*	—	Progressive leukocytosis despite therapy
3	10.7	35.6	—	12.0	287	—	*Morganella morganii* isolated; antibiotics changed to amikacin + piperacillin/tazobactam
34	—	—	—	8.8	—	—	PRBC transfusion for anemia
Follow-up	—	—	—	—	—	—	Follow-up blood culture negative; clinical improvement

CRP, C-reactive protein; WBC, white blood cell count; PRBC, packed red blood cells.

(—): indicates that the parameter was not measured or was unavailable at that time point. Left shift refers to an increased proportion of immature neutrophils on the peripheral blood smear. *The platelet count of 135 × 10⁹/L represents the nadir observed during hospitalization.

**Table 3:** Antibiotic sensitivity test results in our case.

Antibiotic	Disk concentration (µg)	Interpretation
Ciprofloxacin	5	Susceptible
Gentamicin	10	Susceptible
Meropenem	10	Susceptible
Amikacin	30	Susceptible
Cefazolin	30	Susceptible
Trimethoprim/Sulfamethoxazole	1.25/23.75	Susceptible
Ceftriaxone	30	Susceptible
Ceftazidime	30	Susceptible
Piperacillin/Tazobactam	100/10	Susceptible
Cefepime	30	Susceptible
Cefotaxime	30	Susceptible

### Follow up and outcomes

Laboratory abnormalities, such as elevated C-reactive protein, leukocytosis, and thrombocytopenia, may develop over time rather than being present at birth, emphasizing the importance of serial monitoring (15). In our case, although the initial inflammatory markers showed a decreasing trend, with CRP declining from 33 mg/L to 10.7 mg/L, the infant continued to demonstrate concerning clinical findings, including persistent respiratory distress and evolving hematological abnormalities. This highlights that inflammatory markers should not be interpreted in isolation, particularly in extremely preterm infants, where clinical manifestations of infection may be subtle and laboratory responses may not always parallel disease progression. Therefore, antimicrobial decisions should be guided by the overall clinical assessment, microbiological findings, and serial evaluation rather than relying on individual laboratory parameters alone.

### Clinical take away

Further research is needed to better understand the relationship between *in vitro* susceptibility and clinical outcomes in neonates and to establish guidelines for the management of rare Gram-negative infections in this vulnerable population. This highlights the importance of integrating microbiological data with careful clinical assessment when managing neonatal sepsis caused by rare pathogens.

### Review of previously reported cases

There are fewer than 15 reported cases in the literature of neonatal sepsis caused by *Morganella morganii*, most of which are early-onset (5, 9). No clear sex predilection has been observed, and the median age at diagnosis is 1.3 days. Nearly all reported cases involve premature births, with about half delivered via cesarean section (13). Maternal chorioamnionitis was a common antenatal risk factor in five of these cases (7). Many had been exposed to ampicillin or amoxicillin before birth, which may have contributed to the infection (7).

Comparing our case to previously reported instances, several features stand out. First, the initial discordance between gentamicin susceptibility and clinical response is rarely documented. Second, the delayed appearance of hematologic abnormalities emphasizes the necessity of ongoing monitoring, rather than relying solely on initial laboratory results (Table 4). Collectively, these aspects distinguish our case from prior reports and provide a valuable clinical lesson: individualized, response-guided antimicrobial therapy is essential in managing EONS caused by rare pathogens like *Morganella morganii*.

**Table 4:** Reported cases of early neonatal *Morganella morganii* infection.

Author (Year)	Gender	GA (Weeks)	Birth weight (gram)	Maternal risk factors	Antibiotics resistance	Outcome
1. Philip et.al (1997) (16)	Male	40	3,500	No maternal risk	Ampicillin and cefazolin	Survived
2. Rowen et al. (1998) (17)	Male	35	2,180	Chorioamnionitis and funisitis Monthly intramuscular penicillin for rheumatic fever prophylaxis and intrapartum ampicillin and gentamicin.	Ampicillin	Survived
3. Manuel Casanova-roma et.al (2002) (18)	Male	35	2,315	No maternal risk	Ampicillin, amoxicillin, amoxicillin – clavulanic acid, and cefazolin	Survived
4. T. Boussemartet et al. (2004) (19)	Female	30	1,590	Chorioamnionitis	Ampicillin	Survived
5. Yang Chang et.al (2011) (12)	Male	24	532	Premature contractions, Chorioamnionitis	Ampicillin, cefuroxime, cephalothin	Died (at 56 h of age)
6. Ning Chang et al. (2022) (15)	Female	25+6 weeks	690	Cervical incompetence, chorioamnionitis and UTI	Ampicillin, ampicillin-sulbactam, and Cefazolin	Survived
7. Gameiro et al (2023) (5)	Female	28+4 weeks	1,200		Amoxicillin and amoxicillin-clavulanate	Survived
Our case	Male	27 weeks	1,400	UTI	Susceptible to all tested antibiotics	Survived

## Conclusion

This case highlights that antimicrobial susceptibility results alone may not reliably predict clinical outcomes in neonatal sepsis. Although *Morganella morganii* showed *in vitro* susceptible to gentamicin, and empirical therapy was started accordingly, the lack of clinical improvement required a change in antimicrobial therapy. Successful recovery was achieved through treatment adjustments guided by both susceptibility testing and the patient's changing clinical condition. This report emphasizes the importance of combining microbiological data with careful clinical assessment when managing early-onset neonatal sepsis, especially in infections caused by rare or atypical pathogens. Further research is needed to better understand the link between *in vitro* susceptibility and clinical outcomes in neonatal *Morganella morganii* infections.

## Patient guardian perspective

The patient’s family expressed significant emotional distress during the course of the illness and throughout the therapeutic period, particularly due to the critical condition and due to severe symptoms of their newborn and the need for intensive care management. They reported feelings of fear and uncertainty regarding the diagnosis of neonatal sepsis. However, they appreciated the clear communication provided by the medical team, which helped them understand the rationale behind antibiotic courses and its expected benefits. Over time, as the patient’s condition stabilized, the family expressed relief and gratitude for the care provided. They emphasized the importance of continuous updates, emotional support, and involvement in decision-making throughout the treatment process.

## Data Availability

The original contributions presented in the study are included in the article/Supplementary Material, further inquiries can be directed to the corresponding authors.
